# Splenic volume and splenic vein diameter are independent pre-operative risk factors of portal vein thrombosis after splenectomy: a retrospective cohort study

**DOI:** 10.1186/s12893-021-01364-3

**Published:** 2021-10-12

**Authors:** Guillaume Péré, Hubert Basselerie, Charlotte Maulat, Armando Pitocco, Pierrick Leblanc, Antoine Philis, Charles Henri Julio, Géraud Tuyeras, Etienne Buscail, Nicolas Carrere

**Affiliations:** 1 Department of Digestive Surgery, Upper Gastro-Intestinal Tract Surgical Unit, University Hospital of Toulouse, 1 Avenue du Professeur Jean Poulhès, TSA 50032, 31059 Toulouse Cedex 9, France; 2 Department of Radiology, University Hospital of Toulouse, Place du Docteur Baylac, TSA 40031, 31059 Toulouse Cedex 9, France; 3 Unit of Epidemiology, Épidémiologie, Pôle Santé Publique et Médecine Sociale, Faculté de Médecine de Purpan, University of Toulouse III Paul Sabatier, 37 allées Jules Guesde, 31073 Toulouse Cedex, France; 4 Digestive Surgery, Joseph Ducuing Hospital, Hopital Ducuing, 15 rue de Varsovie, 31 300 Toulouse, France; 5 INSERM 1220, IRSD University of Toulouse Paul Sabatier, CHU Purpan, Place du Docteur Baylac, CS 6003931024, Toulouse cedex 3, France

**Keywords:** Splenectomy, Portal thrombosis, Post-operative morbidity, Screening, Lymphoma, Splenic vein

## Abstract

**Background:**

Portal vein thrombosis (PVT) is a common complication following splenectomy. It affects between 5 and 55% of patients undergoing surgery with no clearly defined pre-operative risk factors. The aim of this study was to determine the pre-operative risk factors of PVT.

**Patients and method:**

Single centre, retrospective study of data compiled for every consecutive patient who underwent splenectomy at Toulouse University Hospital between January 2009 and January 2019. Patients with pre- and post-surgical CT scans have been included.

**Results:**

149 out of 261 patients were enrolled in the study (59% were males, mean age 52 years). The indications for splenectomy were splenic trauma (30.9%), malignant haemopathy (26.8%) and immune thrombocytopenia (8.0%). Twenty-nine cases of PVT (19.5%) were diagnosed based on a post-operative CT scan performed on post-operative day (POD) 5. Univariate analysis identifies three main risk factors associated with post-operative PVT: estimated splenic weight exceeding 500 g with an OR of 8.72 95% CI (3.3–22.9), splenic vein diameter over 10 mm with an OR of 4.92 95% CI (2.1–11.8) and lymphoma with an OR of 7.39 (2.7–20.1). The role of splenic vein diameter with an OR of 3.03 95% CI (1.1–8.6), and splenic weight with an OR of 5.22 (1.8–15.2), as independent risk factors is confirmed by multivariate analysis. A screening test based on a POD 5 CT scan with one or two of these items present could indicate sensitivity of 86.2% and specificity of 86.7%.

**Conclusion:**

This study suggests that pre-operative CT scan findings could predict post-operative PVT. A CT scan should be performed on POD 5 if a risk factor has been identified prior to surgery.

## Background

Described in 1895 [1], portal vein thrombosis (PVT) is a common but severe complication following splenectomy [2, 3]. Incidence is reported to be between 5 and 55% in the literature, but a recent meta-analysis published in 2018 suggests an incidence of about 8.1% [4]. This complication is mostly asymptomatic, or symptoms are non-specific, if present [2, 5]. Moreover, PVT can lead to major complications such as digestive ischemia, portal hypertension, cavernoma and even death. Once the diagnosis of PVT is established, an anticoagulant treatment is required before POD 8 to treat PVT [6, 7]. Treatment efficacy and prognosis depend on the length of time to diagnosis. Diagnosis can be carried out by radiological examinations such as US/CT scan with contrast agent and portal sequence. Portal vein thrombosis (PVT) can be diagnosed on CT scans from POD-4 [3].

Nowadays, risk factors are not clearly identified. Authors consider splenomegaly [8] and malignant haematological diseases (lymphoma and myeloproliferative disorder) [9] as responsible for the onset of thrombosis. Recently, some authors suggested splenic vein diameter as another potential and underestimated risk factor [10]. Currently, no screening test based on post-operative imaging is recommended in daily practice despite the incidence of this complication. There are no guidelines on general or individual screening tests for visceral surgeons. Identification of pre-operative risk factors could facilitate earlier diagnosis of PVT with the focus on a high-risk group of patients.

The aim of this study is to determine the pre-operative risk factors (clinical, biological and radiological) of portal vein thrombosis, and to propose screening test criteria for use in daily practice.

## Patients and methods

### Patients and inclusion criteria

We conducted a single centre, retrospective study involving every patient who underwent splenectomy between 1 January 2009 and 1 January 2019 at the University Hospital of Toulouse. The inclusion criteria were consecutive splenectomy with pre-operative and post-operative CT scans and a dedicated portal time assessment. The exclusion criteria were a medical history of splenic, pancreatic or gastric surgery as well as splenic or portal vein thrombosis diagnosed prior to surgery. Patients who underwent splenectomy without a CT scan before or after surgery were excluded. We did not include patients who underwent a CT scan without a contrast agent. Patients screened via ultrasonography before and after surgery were excluded from the study due to a higher variability between observers and measures than CT scan [11].

A CT scan was usually performed on POD 5 to diagnose PVT. The following clinical factors were assessed: medical history, history of thrombosis and antiplatelet or anticoagulant treatment. Indications for splenectomy, per-operative conditions (bleeding, transfusion), laparoscopic-surgery or open surgery, duration of the surgery and splenic weight were analysed. Biological criteria were based on pre-surgical blood tests comprising platelet count, leukocytes, haemoglobin and prothrombin levels. Each patient received the same post-operative prophylactic anticoagulation with subcutaneous Enoxaparin 40 mg, 10 days after surgery.

This study had been designed according to STROCSS criteria for quality improvement [12].

This retrospective study followed French legislation (Loi Bioéthique, November 2016) and CNIL (French Data Protection Authority) guidelines for processing anonymous and retrospective data as well as questionnaires. In accordance with these regulations and given the retrospective nature of the study, patients were not required to give their informed consent for their personal data to be analysed.

This study was declared to the CNIL as an MR-4 procedure (No. 2217213v0), authorised by the local Ethics Committee of the University Hospital of Toulouse. The present study had been registered in “health data hub” system, with the ID: F20210119175811 (www.health-data-hub.fr). It also complies with Declaration of Helsinki guidelines.

### Imaging data

Radiological data from pre-operative CT scans were analysed. Splenic volume was estimated according to splenic dimensions: 0.524 × L × W × T, (where W = maximum width, T = thickness and L = length), and 1 g = 1 mL [13]. The splenic vein diameter measured 2 cm from its termination (just in front of the aorta for reproducible measurements). We documented the diameters of the left gastric and gastro-omental veins and the distance to the splenic hilum. We also identified anatomical variations in order to assess their impact on PVT. Diagnosis of PVT was certified by CT scans performed at POD 5 and analysed by a radiologist and a gastrointestinal surgeon. Pre-operative CT scan data were accepted if agreed by both investigators. Pre-operative and post-operative data were analysed separately in order to blind the outcome.

PVT was defined as thrombosis of the portal vein, possibly associated with thrombosis of the porto-splenic axis as splenic vein, inferior mesenteric vein, superior mesenteric vein or intrahepatic portal vein thrombosis. Patients were assigned to one of two groups depending on the presence or absence of PVT.

Patient with PVT was followed by CT scan (with a dedicated portal time assessment) at POD 30, 90 and 180. Criteria for portal vein recanalization was the disappearance of the clot with a lumen corresponding to the portal vein and the absence of serpiginous vascular channels in porta hepatis according to the European Association for the Study of the Liver (EASL) Clinical Practice Guidelines [11].

### Statistical analysis

Categorical variables were reported as numbers and percentages compared using either Student’s t-test, Pearson Chi2 or the Fisher exact test, as appropriate. The Pearson correlation test was carried out to establish correlation between two continuous variables with linear monotone variation. A result > 0.5 was considered a strong correlation. Multivariate analysis was performed with logistic regression to characterise independent factors. Statistical analysis was performed using STATA 14.2 software (StataCorp LP, College Station, TX, USA). Differences were deemed statistically significant at p < 0.05.

## Results

### General characteristics and surgical data

**Fig. 1:**
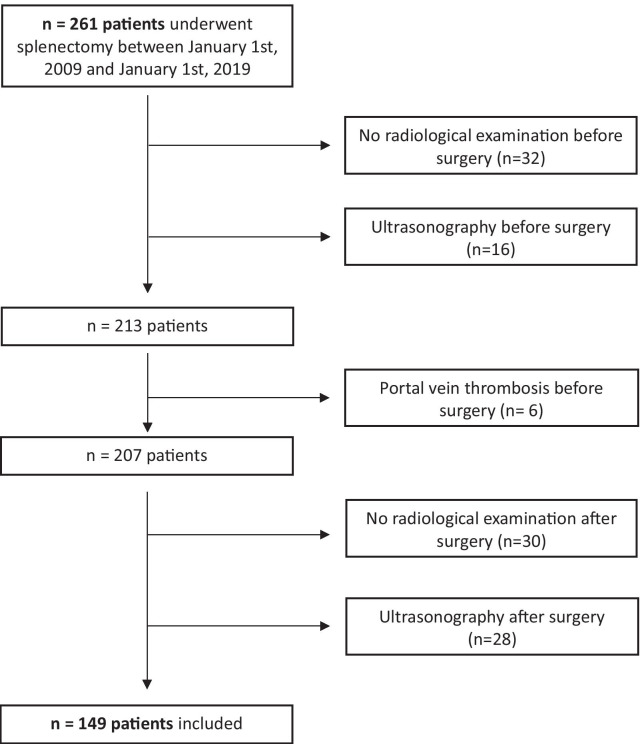
flow chart for the selection of the study population

Two hundred and sixty-one patients underwent splenectomy at the University Hospital of Toulouse from 1 January 2009 to 1 January 2019. Ultimately, 149 patients were enrolled in the study based on the exclusion criteria (Fig. 1). One hundred and twelve patients could not be included due to the absence of a CT scan with contrast agent before or after surgery, or a pre-existent PVT. Thirty patients did not undergo post-surgical screening and ultrasonography was performed for 28 patients. The patients enrolled in this study underwent splenectomy for splenic trauma in 30.9% of cases (n = 46), malignant haemopathy in 26.8% of cases (n = 40) mostly due to lymphoma in 57.5% of cases (n = 27), extended cancer surgery for peritoneal carcinomatosis, pancreatic cancer and gastric cancer in 21.5% of cases (n = 32), immune thrombocytopenia (ITP) in 8.0% of cases (n = 12) and others in 12.8% of cases (n = 19). The characteristics of splenectomy indications are summarised in Table 1. Portal Vein Thrombosis (PVT) was identified from a post-operative CT scan in 29 patients, with an incidence of 19.5% (29/149). The results of the clinical and biological analyses are presented in Table 2. No statistical differences in pre-operative clinical criteria such as gender, age or body mass index were found between the two groups. A medical history of thromboembolic accident or curative anticoagulant therapy and portal hypertension had no impact on the onset of PVT post-splenectomy (p > 0.05). No patient who underwent splenectomy due to splenic trauma developed post-operative PVT (p < 0.001).

**Table 1:** Surgical indications for the study population

Indications for splenectomy	Number (%)
Splenic trauma	46 (30.9)
Malignant haemopathies	40 (26.8)
Lymphoma	27 (18.1)
Myeloproliferative neoplasms	7 (4.7)
Chronic myeloid leukaemia	4 (2.7)
Waldenstrom	2 (1.3)
Extended cancer surgery	32 (21.5)
Peritoneal carcinosis	14 (9.4)
Pancreatic cancer	10 (6.7)
Gastric cancer	8 (5.4)
Immune thrombocytopenia (ITP)	12 (8.0)
Others	19 (12.8)
Benign tumours	10 (6.7)
Sickle cell disease	3 (2.0)
Hydatid cysts	4 (2.7)
Wandering spleen	2 (1.3)

Reasons for undergoing surgery expressed by a number of patients—the values in brackets are percentages.

**Table 2:** Clinical and biological risk factors of patients with or without post-operative PVT

	Patients without PVT n (%) 120 (80.5%)	Patients with PVT n (%) 29 (19.5%)	p-value
Gender F/M^a^	49 (32.9%)/70 (47%)	11 (7.4%)/18 (12.7%)	0.100
Age (years)^b^	50.9 ± 17.0	56.8 ± 17.1	0.070
Body Mass Index (kg/m^2^)^b^	24.8 ± 5.1	25.6 ± 4.7	0.511
Thromboembolic medical history^a^	3 (2.5%)	1 (3.4%)	0.580
Curative anticoagulant therapy^a^	4 (3.3%)	1 (3.4%)	0.670
Portal hypertension^a^	2 (1.7%)	1 (3.4%)	0.480
Indications: ^a^			
Splenic trauma	45 (37.5%)	0 (0%)	** < 0.001**
Malignant haemopathies	24 (20%)	16 (55.2%)	** < 0.001**
Lymphoma	8 (6.7%)	15 (51.7%)	** < 0.001**
Contiguity	27 (22.5%)	5 (17.2%)	0.210
ITP	11 (9.2%)	2 (6.9%)	0.570
Others	13 (10.8%)	6 (20.7%)	0.162
Pre-operative laboratory tests^c^			
Haemoglobin (g/dL)	11.8 (8.8–14.3)	11.3 (9.7–12.8)	0.380
Leukocytes (G/L)	11.9 (6.1–15.4)	15.0 (20.6–5.25)	0.160
Platelets (G/L)	197.1 (175–215)	146.1 (103–188)	0.050
Prothrombin (%)	80.1 (66.2–96.4)	86.4 (80.3–93.8)	0.060
Transfusion^a^	57 (47.5%)	7 (24.1%)	0.051
Per-operative conditions			
OS/LS	101 (84.2%)/19 (15.8%)	25 (86.2%)/4 (13.8%)	0.310
Duration of surgery (min)^b^	130.7 ± 127.9	147.6 ± 129.8	0.541
Splenic weight (g) ^c^	413.7 (307.2–520.3)	1468.6 (1036.9–1900.2)	** < 0.00001**
Post-operative PVT symptoms^a^	64 (53.8%)	9 (31.0%)	**0.028**

Bold values are statistically significant values from the statistical analyses (with p < 0.05)

Results are expressed as ^a^number and percentages in brackets, ^b^mean and standard error of the mean, ^c^number and confidence interval (95% CI) in brackets. OS, Open surgery; LS, Laparoscopic surgery

Malignant haemopathy was identified as a PVT risk factor in 16 cases of thrombosis (55.2%) versus 24 (20%) in the non-PVT group (p < 0.001). In the case of lymphoma, we detected 15 cases (51.7%) with PVT compared to 8 cases (6.7%) without PVT (p < 0.001). Splenectomy as part of extensive cancer surgery was not identified as a significant PVT risk factor with 5 cases of thrombosis versus 27 cases without PVT.

No differences were highlighted in pre-operative laboratory tests (haemoglobin, leukocytes, prothrombin), except for platelets at 146 G/L with a 95% CI (103–188) for the PVT patient group versus 195 G/L with a 95% CI (175–215) and p = 0.05. A lower platelet count appears to be a biological risk factor of PVT post-splenectomy.

Per-operative conditions were not identified as risk factors for the onset of PVT regardless of the technique used. Laparoscopic surgery (LS) does not increase the risk of PVT compared to open surgery, with LS values of 13.8being recorded in the PVT -group versus 15.8% in the non-PVT (p = 0.310). Similarly, the duration of surgery is not a risk factor for the onset of PVT with times of 147.6 min (± 129.8) with PVT versus 130.7 min (± 127.9) without PVT (p = 0.541). The most significant risk factor identified was splenic weight with 1468.6 g, 95% CI (1036.9–1900.2) documented in the PVT group versus 413.7 g, 95% CI (307.2–520.3) in the non-PVT group (p < 0.00001).

Post-operative symptoms were non-specific (nausea, abdominal pains, fever) and non-predictive for PVT in this study. Patients with PVT presented fewer symptoms than their non-PVT counterparts (p = 0.028).

### Radiological and morphological features

The pre-operative radiological assessment (Table 3) highlighted a difference in splenic vein diameter between the two groups. A greater diameter was recorded in the PVT group at 12.3 mm, 95% CI (10.5–14.1) compared to the non-PVT group with 9.02 mm 95% CI (8.5–9.5), p < 0.00001.

**Table 3:** Results of pre-operative CT scan risk factors analysis as a function of the presence of PVT

	Patient without PVT n (%) 120 (80.5%)	Patient with PVT n (%) 29 (19.5%)	p-value
Splenic vein diameter (mm)^b^	9.02 (8.5–9.5)	12.3 (10.5–14.1)	** < 0.00001**
End of the left gastric vein: ^a^			
Portal vein	71 (59.2%)	20 (68.9%)	0.40
Splenic vein	49 (40.8%)	9 (31.1%)	
Left gastro-omental vein diameter (mm)^b^	2.39 (1.5–3.0)	2.47 (1.5–3.0)	0.39
Estimated splenic volume (cm^3^)^b^	520.1 (214–550)	1570.0 (520–2806)	** < 0.0001**

Bold values are statistically significant values from the statistical analyses (with p < 0.05)

Results are expressed as ^a^number and percentages in brackets, ^b^number and 95% confidence interval (95% CI) in brackets

Splenic volume estimated using the formula from pre-operative CT scan findings, increased in the PVT group with a mean of 1570.0 mL 95% CI (520–2806) versus 520.1 mL 95% CI (214–550) in the non-PVT group (p < 0.0001). The Pearson correlation coefficient for splenic weight and estimated splenic volume was 0.97. The estimated pre-operative splenic volume was therefore a relevant method for assessing splenic weight.

The Pearson correlation test performed to establish a link between splenic vein diameter and splenic weight/volume is 0.02 in this instance, indicating the lack of proportional relationship between splenic vein diameter and splenic volume. These two risk factors do not seem to be linked.

### Univariate and multivariate analyses of predictive PVT criteria

Univariate analysis (Table 4) clearly identified a pre-operative splenic vein diameter exceeding 10 mm, with an odds ratio (OR) of 4.92, 95% CI (2.1–11.8), and an estimated splenic weight of over 500 g with an OR of 8.72 95% CI (3.3–22.9). Lymphoma was also identified as a risk factor of post-operative PVT with an OR of 7.39 95% CI (2.7–20.1). Pre-operative thrombocytopenia was associated with an OR of 2.17 95% CI (1.37–2.65).

**Table 4:** Results of univariate and multivariate analysis

	Univariate analysis	Multivariate analysis
Splenic vein diameter > 10 mm	OR = 4.92 (2.1–11.8) (p < 0.0001)	**OR = 3.03 (1.1–8.6) (p < 0.001)**
Splenic weight > 500 g	OR = 8.72 (3.3–22.9) (p < 0.001)	**OR = 5.22 (1.8–15.2) (p < 0.01)**
Lymphoma	OR = 7.39 (2.7–20.1) (p < 0.01)	OR = 1.6 (0.7–3.6) (p < 0.07)
Platelets < 120 G/L	OR = 2.17 (1.1–2.6) (p < 0.05)	OR = 1.2 (0.5–1.7) (p < 0.10)

Bold values are statistically significant values from the statistical analyses (with p < 0.05)

Results of univariate and multivariate analysis of pre-operative risk factors are expressed as Odds Ratio (OR) and 95% confidence interval (95% CI) in brackets

Multivariate analysis (Table 4) identified a splenic vein diameter exceeding 10 mm (OR = 3.03–95% CI 1.1–8.6) and splenic weight (OR = 5.22 95% CI 1.8–15.2) as independent risk factors of portal vein thrombosis post-splenectomy. Lymphoma and thrombocytopenia were not statistically linked to PVT in the multivariate analysis.

### Specificity, sensitivity and predictive values of the univariate analysis risk factors

We performed a screening test based on the presence of two anatomical landmarks identified on the pre-operative CT-scan: splenic vein diameter exceeding 10 mm and splenic weight over 500 mL. The results show optimum sensitivity with the presence of 1 criterion (sensitivity = 0.86). A negative predictive value (NPV) of 0.95 (see Table 5) was recorded in the case of one criterion. Test sensitivity increased to 0.9 for PVT detection in patients presenting malignant haematological disorders. In the lymphoma group, sensitivity reached 100% in the presence of one criterion, and every case of PVT was detected (15/15) (Table 5).

**Table 5:** Analysis of the sensitivity and specificity of splenic vein diameter and splenic weight in the study population

Risk factor	Sensitivity	Specificity	PPV	NPV	PLR	NLR	Accuracy
Splenic weight > 500 g	0.72 (0.53–0.87)	0.78 (0.69–0.85)	0.44 (0.29–0.59)	0.92 (0.85–0.97)	3.22 (2.16–4.81)	0.36 (0.20–0.65)	0.77
Splenic vein diameter > 10 mm	0.72 (0.53–0.87)	0.72 (0.63–0.80)	0.38 (0.25–0.53)	0.91 (0.84–0.96)	2.56 (1.78–3.67)	0.38 (0.21–0.70)	0.72
1 risk factor	0.86 (0.68–0.96)	0.62 (0.53–0.71)	0.36 (0.25–0.48)	0.95 (0.88–0.99)	2.30 (1.75–3.02)	0.22 (0.09–0.55)	0.67
2 risk factors	0.62 (0.42–0.79)	0.87 (0.79–0.92)	0.53 (0.35–0.70)	0.90 (0.84–0.95)	4.66 (2.72–7.97)	0.44 (0.27–0.70)	0.82

Screening test characteristics are expressed as numbers and 95% confidence interval in brackets. PPV, positive predictive value; NPV, negative predictive value; PLR, positive likelihood ratio; NLR, negative likelihood ratio

### Middle and long-term results

After the diagnosis of PVT, every patient received a curative daily sub cutaneous Low Molecular Weight Heparin (LMWH) injection: Tinzaparin 175 UI/kg. We then followed up the treatment with a Vitamin K Antagonist Fluindion for 6 patients (20.7%). Objectives for International Normalized Ratio ranged from two to three. Mean duration of treatment was 107.5 days (± 52.0). CT scan at POD 30, 90 and 180 have been realized to diagnose portal vein recanalization. Results are detailed in Table 6. After POD 180 one patient had a portal cavernoma and one had right portal vein thrombosis. No intestinal infarction has been reported in this cohort.

**Table 6:** Middle and long-term follow-up of patients with PVT

	POD-30	POD-90	POD-180
PVT recanalization	N = 21 patients (72.4%)	N = 25 patients (86.2%)	N = 27 patients (93.1%)
PVT persistence	N = 8 patients (27.6%)	N = 4 patients (13.8%)	N = 2 patients (6.9%)

Post-operative follow-up for 29 patients with portal vein thrombosis treated by anticoagulant therapy 30, 90 and 180 days after splenectomy. Results are expressed as number of patients, percentages in parenthesis. POD, post-operative day; PVT, portal vein thrombosis

## Discussion

### PVT and splenectomy

The literature concerning the incidence of portal vein thrombosis post-splenectomy is heterogeneous. It varies from 5% [8] to 55% [5] and seems to be particularly high in Japanese studies [10, 14, 15]. The differences can be explained by heterogeneous patient selection and the reasons for splenectomy. For instance, portal vein hypertension justified surgery for 76.8% patients undergoing splenectomy (43/56 patients) in a recent Asian study [16]. Portal hypertension (PHT) does not appear to be a PVT risk factor in this investigation, possibly due to an inadequate patient cohort. However, in 2019, Huang et al*.* [17] confirmed a higher risk of PVT in the case of PHT with 32% of PVT versus 9.5% (p < 0.05) of non-PVT in a population with 50% of patients undergoing splenectomy for cirrhosis and portal hypertension. A recent 2018 American meta-analysis from the Mayo Clinic collected data from 1745 patients and calculated a PVT incidence rate of approximately 8.1% [4].

Post-operative clinical symptoms are frequently absent [2, 18] or non-specific (nausea, vomiting, abdominal pain, fever > 38 °C). The literature does not refer to any specific symptom to aid diagnosis. De’Angelis et al*.* [5] confirmed that 49.5% of patients do not present any symptoms of PVT. Our study showed that 69% of patients do not present clinical symptoms of PVT. Clinical examination does not provide the surgeon with any evidence of PVT. Hence the diagnosis has to be based on radiological examination.

In this study, CT scans were performed on POD 5 to diagnose PVT, as thrombosis was clearly identified on POD 4 [3, 8, 14, 19, 20]. PVT can be diagnosed on a CT scan with sensitivity of about 90% and approx. 100% specificity, which is higher than ultrasonography (especially for proximal splenic vein thrombosis) [21]. Treatment is efficient if begun before POD 8. It allows complete regression of the thrombosis and avoids complications such as mesenteric vein thrombosis, digestive ischemia, cavernoma and portal hypertension [6, 7, 18, 22]. Consequently, we recommend a CT scan on POD-5 as a good compromise for detecting post-operative PVT.

### PVT and haematological malignancies

Haematological malignancies such as myeloproliferative disease and lymphoma appear to be a reason for promoting PVT in the literature [3, 9, 19, 23]. Our study does not identify lymphoma as an independent risk factor in the multivariate analysis. Lymphoma and myeloproliferative disorders are usually linked to hypercoagulability because of qualitative and quantitative platelets abnormalities and elevated haematocrit values. Lymphoma is generally responsible for splenomegaly, which is a major risk factor of PVT. This study shows that splenic volume is more predictive than the splenectomy indication in terms of thrombosis (multivariate analysis). The literature does not confirm the role of thrombocytosis or thrombopenia as a PVT risk factor [24]. Thrombopenia is not an independent risk factor in this study but is probably linked to splenic volume [25]. Splenomegaly leads to sequestration syndrome with thrombocytopenia. Following removal of the spleen, the platelet count increases in the bloodstream. The bigger the spleen, the higher the platelet count after surgery. This hypothesis is corroborated by the literature [2, 26], and a recent study by Huang et al. suggests that an increase of 8 in post-operative platelet count could be a PVT risk factor in the first week [17].

### Identified risk factors

This original study is the first of its kind to highlight the role of two independent risk factors in the onset of post-operative portal vein thrombosis, namely splenic vein diameter and splenic volume prior to surgery.

Splenic weight has already been identified and accepted as a PVT risk factor according to the literature [3, 8, 9, 15, 23, 26–28]. However, spleen weight distribution differs quite considerably from one study to the next. For instance, Ikeda et al*.* [15] identified a mean splenic weight of 216 g in a PVT patient group versus 82 g in the non-PVT group (p < 0.05). In our study, splenic weight differed from that presented in the literature and depends on the reasons for splenectomy and the population selection. It suggests that our data are useful for improved extrapolation to the European population. Our study reveals an increased risk of PVT with a splenic weight exceeding 500 g. This cut-off value appears to be both straightforward and appropriate for medical practice with good sensitivity whereas the literature mentions an unreliable cut-off value to screen PVT, such as 650 g according to Stamou et al*.* [8] (about 147 patients), or more recently, 1000 g, according to Ruiz-Tovar et al*.* [26]. In this study, splenic volume was estimated [13] on the basis of 3 spleen dimensions. This simple method for estimating volume correlates well with splenic weight (Pearson coefficient = 0.97). It can be managed independently by a surgeon using the pre-operative CT scan, with no need for 3D reconstruction or any specific software. The splenic length has already been used to predict PVT [5, 19, 29], with an increased risk of PVT over 20 cm (p < 0.05) according to Manoucherhi et al*.* [29].

Our study reveals that splenic vein diameter is another independent risk factor. Few studies have already characterised this anatomical criterion [5, 10, 16, 30]. We measured the splenic vein diameter 2 cm from the splenoportal junction to ensure reproducible measurements and because the vein is perfectly positioned in the axial image of the CT scan. This anatomical site has ever been studied and reported as adequate according to de’Angelis et al*.* [5]. Based on our hypothesis, splenic vein anatomy explains the pathophysiology of portal thrombosis. The interruption or reduction in blood flow through the splenic vein stump decreases the portal flow. This change will be more marked in the case of increased splenic vein diameter and could lead to thrombosis.

We determined a cut-off point of over 10 mm for improved sensitivity in our population. However, few values are highlighted in the literature and these are less appropriate in our population. For example, the cut-off point for splenic vein diameter varies from 8 to 14 mm from one study to the next [5, 10, 16, 30]. Splenic vein diameter and splenic weight are not correlated values. A splenic vein diameter exceeding 10 mm was used to diagnose 13.8% (n = 4) of PVT in our population whereas the splenic volume was below 500 mm^3^.

### A screening test based on pre-operative CT scan landmark

Our findings with independent pre-operative risk factor cut-off values adapted for daily practice could improve the early detection of PVT. To our knowledge, our screening criteria generate the highest sensitivity and specificity for PVT screening in the literature. The presence of one anatomical landmark (splenic vein diameter or splenic volume) reached a sensitivity of about 0.86 in this cohort coupled with a negative predictive value (NPV) of about 0.95. We assume that these criteria have to be highlighted on a pre-operative CT scan, in order to direct the prescription of a post-operative CT scan if one criterion is present. The presence of two risk factors ensures better specificity of approx. 0.87. In the case of lymphoma, this screening test records sensitivity of around 1 with every case of PVT being detected in this group (15/15).

## Limitation of the study

The main limitation of our study is its single centre, retrospective design. However, this study analyses data from 149 patients. Another limitation of our study in terms of calculating the incidence rate is the number of patients excluded (n = 112). We did not enrol patients who underwent pre-operative ultrasonography because the radiologist did not measure splenic vein diameter and splenic volume. We analysed CT data with regard to the few variations between observers (lower than US). The majority of patients without a pre-operative CT scan underwent splenectomy for splenic trauma or immune thrombocytopenia (ITP). Indeed, we did not perform a systematic post-operative CT scan based on published data as these conditions do not promote PVT [31, 32]. ITP is not a risk factor, as demonstrated in a recent study published in 2019, which detected only 2 cases of PVT in a cohort of 109 patients who underwent splenectomy [31]. We reported portal vein thrombosis in 195% of cases (29/149).

### Purpose

Nowadays, there is no consensual recommendation for screening test after splenectomy. Indication for a systematic screening test is debated in the literature. A systematic test for every patient is sustained by several studies [5, 19], justified by the morbi-mortality of PVT and the efficacy of anticoagulant therapy if initiated before POD 8. A screening test based on the presence of risk factors such as splenomegaly or malignant haemopathy, appears to be more justified for several authors [7, 9, 20, 23, 26, 28].

Our study suggests that a CT-scan should be performed on POD 5 following splenectomy in the presence of pre-operative risk factors such as haematological malignancies (lymphoma), an estimated splenic volume exceeding 500 mL and a splenic vein diameter over 10 mm.

Curative anticoagulant treatment is introduced as soon as possible after diagnosis. It should be initiated before POD 8 to promote efficacy and reduce sequelae.

## Conclusion

This study identifies splenic vein diameter and splenic weight as two independent pre-operative risk factors for portal vein thrombosis following splenectomy. In the case of splenic vein diameter > 10 mm or splenic weight > 500 g, a CT-scan must be performed on POD 5. This data could assist surgeons in the earlier detection and treatment of PVT. Further investigations such as prospective studies are required in order to corroborate these criteria.

## Data Availability

The datasets used and/or analysed during the current study are available from the corresponding author on reasonable request.

## References

[1] Delatour HB (1895). Thrombosis of the mesenteric veins as a cause of death after splenectomy. Ann Surg.

[2] Rattner DW, Ellman L, Warshaw AL (1960). Portal vein thrombosis after elective splenectomy. An underappreciated, potentially lethal syndrome. Arch Surg Chic Ill..

[3] Chaffanjon PC, Brichon PY, Ranchoup Y, Gressin R, Sotto JJ (1998). Portal vein thrombosis following splenectomy for hematologic disease: prospective study with Doppler color flow imaging. World J Surg.

[4] Tsamalaidze L, Stauffer JA, Brigham T, Asbun HJ (2018). Postsplenectomy thrombosis of splenic, mesenteric, and portal vein (PST-SMPv): a single institutional series, comprehensive systematic review of a literature and suggested classification. Am J Surg.

[5] de Angelis N, Abdalla S, Lizzi V, Esposito F, Genova P, Roy L (2017). Incidence and predictors of portal and splenic vein thrombosis after pure laparoscopic splenectomy. Surgery.

[6] Ponziani FR, Zocco MA, Campanale C, Rinninella E, Tortora A, Di Maurizio L (2010). Portal vein thrombosis: insight into physiopathology, diagnosis, and treatment. World J Gastroenterol.

[7] van’t Riet M, Burger JW, van Muiswinkel JM, Kazemier G, Schipperus MR, Bonjer HJ (2000). Diagnosis and treatment of portal vein thrombosis following splenectomy. Br J Surg.

[8] Stamou KM, Toutouzas KG, Kekis PB, Nakos S, Gafou A, Manouras A (1960). Prospective study of the incidence and risk factors of postsplenectomy thrombosis of the portal, mesenteric, and splenic veins. Arch Surg Chic Ill.

[9] Svensson M, Wirén M, Kimby E, Hägglund H (2006). Portal vein thrombosis is a common complication following splenectomy in patients with malignant haematological diseases. Eur J Haematol.

[10] Danno K, Ikeda M, Sekimoto M, Sugimoto T, Takemasa I, Yamamoto H (2009). Diameter of splenic vein is a risk factor for portal or splenic vein thrombosis after laparoscopic splenectomy. Surgery.

[11] European Association for the Study of the Liver. Electronic address: easloffice@easloffice.eu. EASL Clinical Practice Guidelines: Vascular diseases of the liver. J Hepatol. 2016;64:179–202. 10.1016/j.jhep.2015.07.040 26516032

[12] Agha R, Abdall-Razak A, Crossley E, Dowlut N, Iosifidis C, Mathew G (2019). STROCSS 2019 Guideline: strengthening the reporting of cohort studies in surgery. Int J Surg Lond Engl.

[13] Yetter EM, Acosta KB, Olson MC, Blundell K (2003). Estimating splenic volume: sonographic measurements correlated with helical CT determination. AJR Am J Roentgenol.

[14] Ikeda M, Sekimoto M, Takiguchi S, Yasui M, Danno K, Fujie Y (2007). Total splenic vein thrombosis after laparoscopic splenectomy: a possible candidate for treatment. Am J Surg.

[15] Ikeda M, Sekimoto M, Takiguchi S, Kubota M, Ikenaga M, Yamamoto H (2005). High incidence of thrombosis of the portal venous system after laparoscopic splenectomy: a prospective study with contrast-enhanced CT scan. Ann Surg.

[16] Kuroki T, Kitasato A, Tokunaga T, Takeshita H, Taniguchi K, Maeda S (2018). Predictors of portal and splenic vein thrombosis after laparoscopic splenectomy: a retrospective analysis of a single-center experience. Surg Today.

[17] Huang D, Tao M, Cao L, Wang X, Zheng S, Cao Y (2019). Risk factors and anticoagulation effects of portal vein system thrombosis after laparoscopic splenectomy in patients with or without cirrhosis. Surg Laparosc Endosc Percutan Tech.

[18] Krauth M-T, Lechner K, Neugebauer EAM, Pabinger I (2008). The postoperative splenic/portal vein thrombosis after splenectomy and its prevention–an unresolved issue. Haematologica.

[19] Bouvier A, Gout M, Audia S, Chalumeau C, Rat P, Deballon O (2017). Routine screening of splenic or portal vein thrombosis after splenectomy. Rev Med Interne.

[20] Tran T, Demyttenaere SV, Polyhronopoulos G, Séguin C, Artho GP, Kaneva P (2010). Recommended timing for surveillance ultrasonography to diagnose portal splenic vein thrombosis after laparoscopic splenectomy. Surg Endosc.

[21] Bradbury MS, Kavanagh PV, Chen MY, Weber TM, Bechtold RE (2002). Noninvasive assessment of portomesenteric venous thrombosis: current concepts and imaging strategies. J Comput Assist Tomogr.

[22] Qi X, Bai M, Guo X, Fan D (2014). Pharmacologic prophylaxis of portal venous system thrombosis after splenectomy: a meta-analysis. Gastroenterol Res Pract..

[23] Loring LA, Panicek DM, Karpeh MS (1998). Portal system thrombosis after splenectomy for neoplasm or chronic hematologic disorder: is routine surveillance imaging necessary?. J Comput Assist Tomogr.

[24] Lee DH, Barmparas G, Fierro N, Sun BJ, Ashrafian S, Li T (2015). Splenectomy is associated with a higher risk for venous thromboembolism: a prospective cohort study. Int J Surg Lond Engl..

[25] Qian Y-Y, Li K (2017). The early prevention and treatment of PVST after laparoscopic splenectomy: a prospective cohort study of 130 patients. Int J Surg Lond Engl.

[26] Ruiz-Tovar J, Priego P (2017). Portal vein thrombosis after splenic and pancreatic surgery. Adv Exp Med Biol.

[27] Patel AG, Parker JE, Wallwork B, Kau KB, Donaldson N, Rhodes MR (2003). Massive splenomegaly is associated with significant morbidity after laparoscopic splenectomy. Ann Surg.

[28] Winslow ER, Brunt LM, Drebin JA, Soper NJ, Klingensmith ME (2002). Portal vein thrombosis after splenectomy. Am J Surg.

[29] Manouchehri N, Kaneva P, Séguin C, Artho GP, Feldman LS (2016). Screening for thrombophilia does not identify patients at risk of portal or splenic vein thrombosis following laparoscopic splenectomy. Surg Endosc.

[30] Kinjo N, Kawanaka H, Akahoshi T, Tomikawa M, Yamashita N, Konishi K (2010). Risk factors for portal venous thrombosis after splenectomy in patients with cirrhosis and portal hypertension. Br J Surg.

[31] Tastaldi L, Krpata DM, Prabhu AS, Petro CC, Haskins IN, Perez AJ (2019). Laparoscopic splenectomy for immune thrombocytopenia (ITP): long-term outcomes of a modern cohort. Surg Endosc.

[32] Parker HH, Bynoe RP, Nottingham JM (2003). Thrombosis of the portal venous system after splenectomy for trauma. J Trauma.

